# Successfully sustaining sex and gender issues in undergraduate medical education: a case study

**DOI:** 10.1007/s10459-016-9742-1

**Published:** 2017-01-03

**Authors:** Francisca van der Meulen, Cornelia Fluit, Mieke Albers, Roland Laan, Antoine Lagro-Janssen

**Affiliations:** 1 Department of Primary and Community Care, Gender and Women’s Health, Radboud University Medical Center, Postbox 9101, 6500 HB Nijmegen, The Netherlands; 2 Radboudumc Health Academy, Research in Learning and Education,, Radboud University Medical Center, Postbus 9101, 6500 HB Nijmegen, The Netherlands

**Keywords:** Sex and gender, Implementation, Sustainability, Undergraduate medical education

## Abstract

Although several projects have addressed the importance of gender health issues in medical education, the sustainability of change efforts in medical education has rarely been addressed. Understanding the possible facilitators or barriers to sustainability may help to develop future interventions that are effective in maintaining gender health issues as a topic in medical curricula. The aim of this study is to provide a longitudinal evaluation of changes regarding gender health issues that occurred in the past decade and the factors that influenced this process. The coursebooks of eight theoretical courses of the Nijmegen medical curriculum were screened on the basis of criteria for an integrated gender perspective in medical education. To assess the sustainability of gender health issues, the screening results from 2014 were compared with those of a similar project in 2005. In addition, open interviews were conducted with eight coordinators to identify facilitators and barriers influencing the sustainability of gender health issues. Analysis showed that, over the past decade, the implementation of gender health issues was mainly sustained and additional changes were made, resulting in an ongoing gender perspective in the Nijmegen medical curriculum. The coordinators mentioned several factors that influenced the sustainability of implementation in medical education: coordinators’ and teachers’ gender-sensitive attitude, competing demands, the presence of sex and gender in learning objectives, examinations and evaluation, organizational support and curriculum revisions. Our findings suggest that, in implementing sex and gender in medical education, medical faculties need to focus on top-down support in incorporating sex and gender into core objectives and time spent on incorporating sex and gender into medicine, and on the continuous training of teaching staff.

## Introduction

The value of integrating sex and gender into medicine has been increasingly acknowledged over the past decades (Berg et al. 2015; Schenck-Gustafsson et al. 2012; WHO 2006). Several publications on topics such as pharmacotherapy, cardiovascular diseases, developmental disorders and doctor-patient communication suggest that sex and gender make a difference at distinct stages of prevention, diagnosis, treatment and prognosis (Baggio et al. 2013; Bartley and Fillingim 2013; Dielissen et al. 2011; Janssen and Lagro-Janssen 2012; Mosca et al. 2011; Soldin and Mattison 2009; Wijngaarden-Cremers et al. 2014).

Although the binaries can be problematic because of their inevitable interaction, sex differences are generally understood as relating to the biological attributes that distinguish male from female, and gender differences to socially constructed roles of men and women, involving masculinity and femininity (Oliffe and Greaves 2012). Gender health issues are defined as diseases or conditions unique to, more prevalent or more serious in men or in women, including diseases for which etiology, manifestations, risk factors, courses or interventions differ in men and in women (Verdonk et al. 2009).

It is important, therefore, that medical students learn to be aware of sex and gender differences in biomedical and social contexts and understand the role of their own gender in their profession as doctors. For this to occur, addressing health issues from a gender perspective must be a goal of medical education. In a medical curriculum with an integrated gender perspective, students have to learn about the effects of gender health issues in medical care and develop practical skills for use in their future work. To make sure doctors apply a gender perspective in their patient care, they need to have the ability to perceive existing gender differences, issues and inequalities and incorporate these into strategies and actions, also known as gender sensitivity (Nobelius and Wainer 2004).

In 2006, the World Health Organization initiated a meeting to review initiatives for integrating sex and gender in curricula and lessons learned (WHO 2006). Multiple universities have appointed special departments to raise awareness, conduct research and teach medical students about gender health issues. Subsequently, several medical committees and councils throughout the United States, Canada, Australia and Europe have undertaken multiple projects to address the importance of a gender perspective in patient care. The Canadian Institute of Gender and Health (IGH) can be seen as a pioneer when it comes to the promotion of research on sex, gender and health (Coen and Banister 2012). The IGH has developed many educational materials on sex and gender issues which can be used in medical education. In addition, the IGH developed online training modules for healthcare researchers. In Europe, from 2010 to 2012, seven universities worked together under the heading EUGIM (European Curriculum in Gender Medicine) to address awareness of gender health issues in medical training programs. To this end, they developed a Gender Medicine training module, which can be used as a course in Bachelor’s and Master’s programs.

In the Netherlands, the Dutch Blueprint of 2009, listing learning objectives for medical students, also requires that faculties educate students to gain an understanding of sex and gender differences in health and illness and become aware of their own position, socialization, norms and values and take these into account when working in healthcare (Van Herwaarden et al. 2009). A 2005 project already described the factors that played a role in the development of an integrated gender perspective in the medical program, and of the initial incorporation of sex and gender into medical education at a Dutch medical faculty (Verdonk et al. 2005).

### Sustainability of implementation projects

While the initial implementation of gender health issues into medical education has been reported in several studies over the past decade (Brewster et al. 2015; Buchanan et al. 2005; Cheng and Yang 2015; Ludwig et al. 2015; Verdonk et al. 2005; WHO 2006), the long-term maintenance of these projects remains underexposed. Although several factors may help to create conditions that facilitate initial implementation, their presence or influence may diminish over time (Stirman et al. 2012). Therefore, many initiatives that are initially successful fail to become part of organizations (Stirman et al. 2012; WHO 2006). It proves to be difficult to embed implementation projects in education on a structural basis, causing innovations not to be securely realized in curricula or to be abandoned (Sleegers et al. 2014). To ensure project maintenance, new practices must be integrated into routines and embedded in the organization (Grol et al. 2007).

Studies on implementation projects beyond medical education document multiple facilitators that should be taken into account when aiming for maintenance, such as quality control and the adaptability of an intervention to its context and to the organization’s future wishes (Celik et al. 2009; Glind et al. 2015; Grol et al. 2007). However, little is known about the sustainability of implementation projects on gender health issues in medical education. Similar to these studies in healthcare, we need to ensure the permanence of implementation efforts in medical education, by assessing the sustainability of gender health issues over the years.

The 2005 project gave us the unique opportunity both to evaluate the sustainability of the incorporated gender health issues and to get insight into factors that played a role in that process over a ten-year period. In this article, we evaluate the sustainability of the 2005 implementation project in the Bachelor’s curriculum over the past decade, and identify facilitating and impeding factors for sex and gender integration in the long term. Understanding possible facilitators and barriers to change may help to develop effective future interventions on gender health issues that become successfully anchored in medical education.

## Method

We used descriptive methods to indicate the maintenance of the adjustments of the 2005 project, and the presence of the criteria of an integrated gender perspective in the medical curriculum in 2014 (Box 1). Qualitative research methods were used to assess facilitating and impeding factors in the sustainability of implementation over a ten-year period. Where applicable, we applied the consolidated criteria for reporting qualitative research (COREQ) (Tong et al. 2007).

**Box 1:** Criteria of an integrated gender perspective in the medical curricula

1. Students are able to recognize and explain sex/gender differences with regard to issues, such as pharmacotherapy, urinary tract infections and other micturition complaints, sexual abuse and violence, partner violence, cardiovascular disease and communication 2. These sex/gender differences are included in the objectives of the education received by students 3. Students have received education that focused on both biomedical and psychosocial differences 4. Students have received education on sex/gender differences over the course of several study years (minimum 2 years) 5. Students have received education in which specific attention was paid to sex/gender differences in at least 6–8 courses (of 2–4 weeks) in the core curriculum 6. Students have been given the opportunity to take one extra optional course on sex/gender, whether or not in combination with ethnicity/culture	

### The 2005 project

**Fig. 1:**
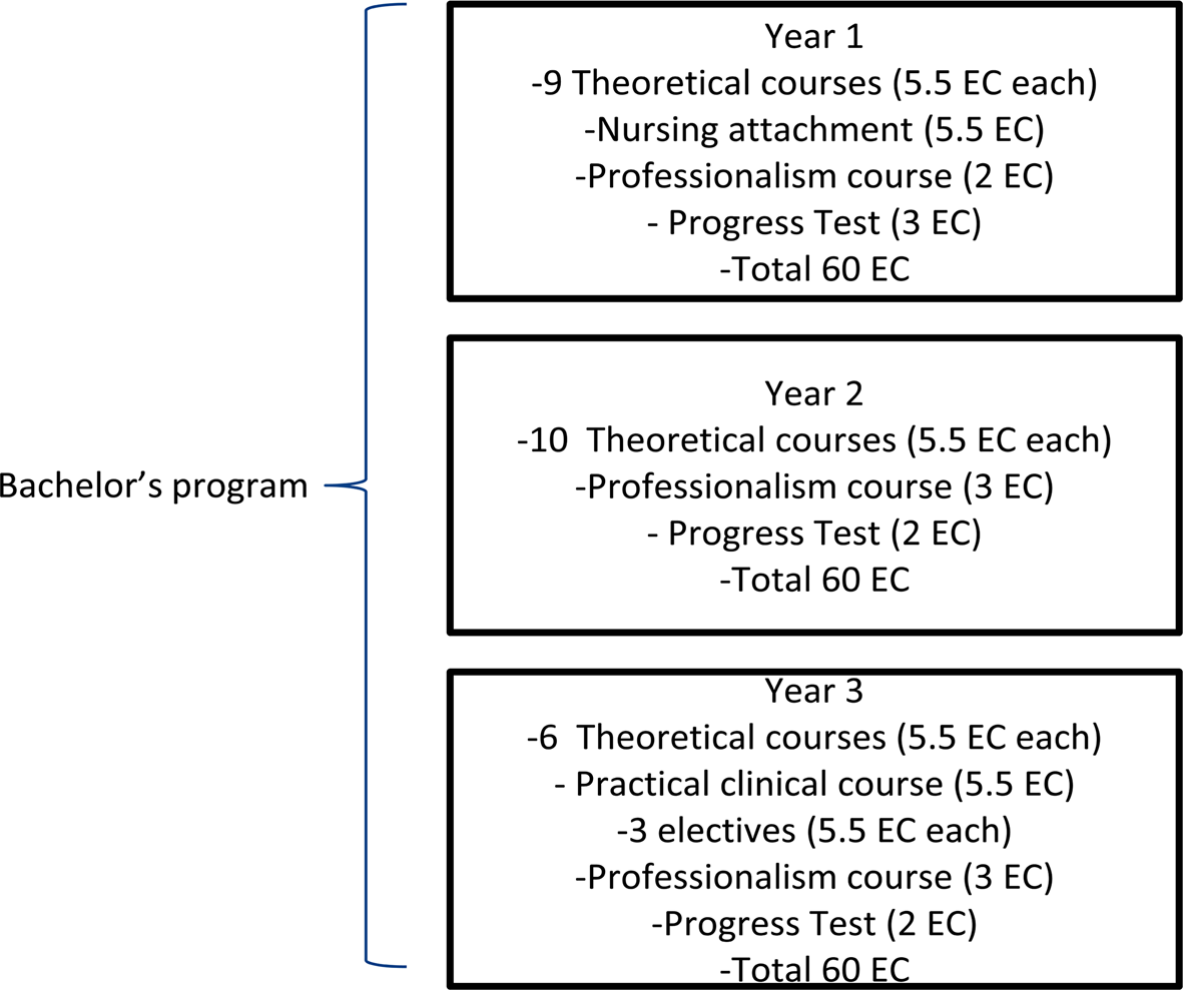
Bachelor’s program at the Radboud university medical center, based on de Visser et al. (2016)

Before the start of the 2005 project, the Bachelor’s program in the Nijmegen medical curriculum (Fig. 1) was screened for the presence of sex and gender issues. Attention to sex and gender issues was fractured, and there were gaps in various fields of knowledge and professional behavior (Sanden et al. 1999). Besides gender issues, some established biological differences between men and women—for instance, in coronary heart disease—were not present in the curriculum either.

Therefore, an implementation project was conducted in 2005 (Verdonk et al. 2005). Based on the literature and international projects, the researchers made a list of characteristics that ought to be present for the integration sex and gender issues into medical education to be considered successful. Where sex or gender issues were absent, adjustments were proposed to have them incorporated, and consultations were held with coordinators to draw their attention to sex and gender issues and to discuss how these adjustments could be implemented. With a total of fourteen proposed adjustments having been implemented according to the criteria of an integrated gender perspective in medical education (Box 1), it was concluded that the implementation project had been successful.

### Describing and comparing curricula

Following the same procedure as in 2005, the criteria formulated in the previous study (Box 1) were used to analyze the Nijmegen medical curriculum for gender health issues. We selected seven theoretical courses in the Nijmegen Bachelor’s curriculum, identical to 2005 (criterion 5). Due to curricular revisions, one 2005 course had been rescheduled to the Master’s program, and a comparable course, therefore, was included in our 2014 screening. These eight courses were spread over 3 years (criterion 4). We indicated whether students had been given the opportunity to take one extra optional course on sex/gender (criterion 6).

The eight 2014 coursebooks were analyzed for the presence of sex and gender differences in several topics (criterion 1). Sex- and gender-specific issues in each coursebook were counted by two researchers (FvdM and MA), who performed line-by-line coursebook analysis. These researchers noted when sex or gender differences were discussed and described their educational format, i.e., objective, lecture or assignment (criterion 2), and their orientation, i.e., biomedical (sex) or psychosocial (gender) (criterion 3).

Subsequently, the 2014 screening results were compared to the 2005 results to assess whether the changes had been sustained or had disappeared over time. In addition, results were compared to indicate which sex- and gender-related adjustments had been added between 2005 and 2014. As one course had been rescheduled to the Master’s program, we indicated changes in the comparable course by comparing the 2005 and the 2014 versions of the coursebook.

### Interviewing course coordinators

The findings from the 2014 coursebook screening were used as discussion points in open interviews with the coordinators of the eight selected courses. These eight coordinators were teachers, appointed to develop and coordinate the course by chairing the teaching committee of the course, who played an important role in the content of the course and managed course content coherence. Coordinators were approached by e-mail, explaining the subject of the research. All coordinators were willing to participate in the study.

The interviews took place between September 2014 and December 2014 at the Radboud university medical center and took 30–45 min. All interviews were guided by one researcher (either FvdM, MSc, educationalist, or MA, PhD) and observed by an experienced senior researcher (AL, PhD MD, Professor of Gender and Women’s Health), who clarified questions if necessary. The researchers had neither personal nor professional relations with any of the coordinators. The interviews were not recorded to avoid the coordinators’ reluctance to participate or to discuss openly. Full notes were presented to them for checking. The quotations from these interviews that were selected for inclusion in this article were translated from the Dutch into the English language.

The opening question was: What facilitating and impeding factors have you faced when maintaining sex and gender issues in your course during the last decade? Coordinators were encouraged to give examples with the arguments they made. No repeat interviews were held.

To analyze the data, content analysis was conducted. The systematic examination of transcripts involved two members of the research team (FvdM and MA). The researchers read and re-read the interview notes independently and met regularly to discuss the subjects and interpretations. By using axial and selective coding, codes were attributed to text segments. Codes referring to the same phenomenon were grouped into categories, and significant themes and key concepts were made explicit and arranged. Categorization differences were discussed with the other members of the research team until agreement was reached. No important disagreements emerged. These themes formed the structure of the final result.

The study protocol was approved by the Ethical Review Board of the Netherlands Association for Medical Education (NVMO-ERB). The Board decided that the study was carried out in accordance with the rules pertaining to the review of research ethics committees and informed consent (NERB-file number 539, 15–07–2015). Participation in this study was voluntary, and participants were not paid for their participation. The notes of the interviews were only accessible to the researchers. No comments were attributed to any participant.

## Results

### Sex and gender issues in the coursebooks: a decade later

Out of the thirteen adjustments we were able to compare, a total of eight had been sustained over the past decade (Table 1). Specifically, biomedical differences between men and women had been retained, whereas some psychosocial differences had disappeared. For example, in one course, lectures about sex differences in digestive tract disorders had been sustained, whereas a lecture on gender-related violence had disappeared. Moreover, several adjustments that had not been implemented in 2005 had been added between 2005 and 2014, such as the toxicity of medication use in pregnancy and sex differences in the prevalence of eating disorders, depression and schizophrenia. According to criterion 3, both biomedical and psychosocial gender differences were still addressed.

**Table 1:** Overview of the sustainability of gender issues 2005–2014

Course	Year	Adjustment	Orientation
Sustained			
Regulation and integration 1	1	Sex as a factor of variability in reactions to medication	Biomedical
Circulation 2	2	Sex related to presentation of chest pain, coronary diseases	Biomedical
	2	Sex/gender-specific items in self-study assignment and/or working group	Biomedical/psychosocial
	2	Gender stereotyping towards patients	Psychosocial
Metabolism 2	2	Gender/sex differences; differences in prevalence and cause in patient cases	Biomedical/psychosocial
	2	Gender/sex differences in diagnosis and treatment of upper abdominal complaints, paying special attention to sexual abuse	Biomedical/psychosocial
	2	Sex differences in digestive tract disorders, particularly constipation and fecal incontinence	Biomedical
	2	Contraceptive and infertility aspects in Crohn’s disease	Biomedical
Disappeared			
MPD 1: Doctor and patient	1	Gender as a psychosocial factor in the bio-psychosocial model	Psychosocial
MPD 4: Doctor and healthcare	3	Gender in the consulting room	Psychosocial
	3	Gender and socio-economic health differences	Psychosocial
	3	Gender-specific healthcare as a form of healthcare	Psychosocial
Metabolism 2	2	Abdominal pain as connected with sexual abuse; female victim invited to the lecture	Biomedical/psychosocial
Added between 2005–2014			
MPD 1: Doctor and patient	1	Sex differences in the prevalence of several diseases: breast cancer, COPD, CVS and epilepsy	Biomedical
Regulation and integration 1	1	Toxicity of medication use in pregnancy	Biomedical
Water and salt metabolism 2	2	Sex differences in urinary tract infections and incontinence	Biomedical
Mental problems	3	Sex differences in prevalence of eating disorders, depression and schizophrenia	Biomedical
Reproduction	3	Sex/gender differences in contraception, sexuality, sexual problems, STDs and sexual identity	Biomedical/psychosocial
	3	Differences in approach to various age groups and sex on the basis of urinary tract infections	Biomedical

*MPD* medical professional development

Sex- and gender-specific issues such as pharmacotherapy, cardiovascular disease, urinary tract infections, reproduction and communication were also present in the 2014 medical curriculum (criterion 1). Seven courses (criterion 5), spread over several study years (criterion 4), dealt with sex and gender differences, an increase of one course compared to 2005. Gender competence became a learning objective in four courses (criterion 2), defined as:

The students are required to be able to describe and analyze the influence of society and culture on health and illness and on the clinical decision process, particularly with regard to gender, ethnic diversity, multiculturalism and medical-ethical views. (learning objective in all Medical Professional Development courses).

In 2014, two sex- and gender-related electives were offered to third-year Bachelor’s students: a Dutch course on ‘Gender, sexuality and ethnicity’ which had already been present in 2005 and an international course on ‘Gender, disease and healthcare’ (criterion 6). The main objectives of these courses were to teach students how sex and gender influence prevention, diagnosis, therapy and prognosis of specific health problems, such as gender-based violence, sexually transmitted infections (STIs) and developmental disorders. The influence of gender in communication, coping styles, patient autonomy and the doctor’s professional role was also discussed, as well as how to bring these skills into practice.

### Facilitating and impeding factors for sustainability

We interviewed five male and three female coordinators (age 42–61 years), one of whom had already been a coordinator in 2005. The coordinators mentioned several facilitating or impeding factors in the sustainability of gender health issues, which could be grouped into the following themes: coordinators’ and teachers’ gender-sensitive attitude, competing demands, the presence of sex and gender in learning objectives, examinations and evaluations, organizational support and curriculum revisions.

### Coordinators’ and teachers’ gender-sensitive attitudes

The coordinators’ own experiences with sex and gender differences as doctors in daily practice was mentioned as one of the sustainability factors; they were all convinced that a gender-sensitive attitude was necessary for good clinical practice, and they all considered themselves to be sex- and gender-minded and showed knowledge of sex and gender issues within their domain. As they acknowledged the importance of gender sensitivity, they also felt it was obvious that sex and gender issues should be maintained in medical education.

Sex and gender issues are inherent in the topic of the abdomen. You have to pay attention to sex and gender differences in complaints and diseases; you cannot deny them. (P1, male).

One coordinator added that the presence of enthusiastic medical teachers with sex and gender expertise was vital for sustainability. Not only are enthusiastic teachers motivated to keep discussing sex and gender in their own lectures, but they can also inspire other teachers. Another coordinator mentioned that knowledge of sex and gender issues should be discussed more openly and explicitly with colleagues to show them the added value of sex and gender differences in their specialism.

I’m sure that the teachers involved in my course have implicit knowledge of sex and gender issues. Nevertheless, I do think that it would be helpful for the development of educational materials to discuss sex and gender issues more explicitly in staff meetings. (P2, male)

He also mentioned that the lack of teacher participation in course development played a negative role. In his view, most teachers did not show up in staff meetings and only felt responsible for their own specific part of the course. This lack of motivation and involvement of professionals in educational development made sex and gender issues hard to sustain.

Education at a university medical center is considered as a mandatory chore. It’s difficult to arrange meetings with the whole coordinating staff, and some teachers are unmotivated. (P2, male)

### Competing demands

Even if project aims had been explicitly accepted, the coordinators felt that there was limited actual scope for them to develop new educational materials and keep up with expanding knowledge of gender health issues. All coordinators mentioned they experienced competing research, education and care demands.

It’s all too much for me. I want to improve my teaching and have numerous ideas about it (sex/gender), but I simply cannot pay any attention to it because I don’t have time to do so. (P3, female)

The presence and availability of accessible educational materials and scientific literature, therefore, were mentioned as important facilitating factors in implementing and sustaining gender health issues. Some coordinators mentioned that access to the Nijmegen digital knowledge center, providing educational tools and literature on sex and gender issues, had been very helpful.

The presence of appealing educational materials such as videos and assignments about borderline personality disorder and gender differences in medication is useful for integrating sex and gender into the course. (P3, female)

### Incorporation into learning objectives, examinations and evaluations

Two coordinators recommended that courses should have well-formulated learning objectives illustrating the relevance of sex and gender issues for the students’ future practice as doctors. Additionally, one coordinator noticed that sex and gender issues should also be incorporated into the students’ examinations to make sure that students meet these learning objectives. Objectives and examinations on sex and gender needed to be firmly anchored in course evaluations, in order to prevent a shift in focus.

Sex and gender are also aspects that must be evaluated and that should, therefore, be included in objectives and in evaluation and monitoring. That would prevent it from vanishing when another topic, such as evidence-based medicine, comes along that must be taught. (P1, male)

When the educational institute centralizes a new theme, such as evidence-based medicine, or as the correspondent mentioned later, care for the elderly or ethics, a theme such as sex/gender may be put on a backburner. Incorporation of gender health issues, however, also depended on the timing of courses. Due to the Bachelor’s program’s theoretical approach, two coordinators indicated that the suggested adjustments did not fit course objectives over time. They felt that it would be confusing for novice students to discuss sex and gender early on in the program. Sex and gender differences, therefore, should be introduced at a later stage in the curriculum. Moreover, as courses must contain a variety of topics and coordinators had to deal with competing priorities, this sometimes led to a changing shift of topics.

A ‘Gender-Sensitive Healthcare’ video, with a chapter on depression, is still present in the course, but now the focus has changed to illustrate a bio-psychosocial approach, instead of illustrating gender-sensitive communication. (P3, female)

### Organizational support and revisions

Four coordinators mentioned that organizational support was crucial in maintaining a gender perspective in medical education. One coordinator said that, to integrate sex and gender issues successfully into medical education, an implementation project should fit the top-down vision of priorities for incorporation into medical education.

To ensure sustainability, you need a strongly committed educational institute with authority within reach, such as the one in Nijmegen. A committed educational management team will enforce a structural evaluation process of the medical curriculum, which benefits the maintenance of sex and gender issues. (P1, male)

Six coordinators thought the revision of the Bachelor’s program, launched in September 2015, might be facilitating. Two coordinators explicitly said that the revision facilitated them to embed gender health issues more firmly at the start of the curriculum, as it allowed them to discuss what topics needed to be anchored in the curricula and in what way. One coordinator thought that it was easier now to incorporate new courses without fragmentation, as the value of sex and gender was widely acknowledged in medicine nowadays, and educational materials on sex and gender were available. Another coordinator nevertheless suggested that the success of implementation could easily be undone in a curriculum revision as competition with other topics would only increase.

## Discussion

The most important finding of our study is that the majority of the adjustments made in the 2005 Bachelor’s curriculum had been sustained over the past decade. Moreover, although some issues had disappeared in 2014, several new issues had been added, resulting in an overall increase of sex and gender issues that had been brought to students’ attention over the past decade. When we applied the criterion framework, the 2014 curriculum has successfully maintained its gender perspective. Despite previous studies showing that it is difficult to embed changes securely in education (Bland et al. 2000; Sleegers et al. 2014), we identified several factors that have influenced this successful sustainability over the years. In line with the implementation literature in healthcare, we found several factors at different levels to be important (Celik et al. 2009; Glind et al. 2015; Grol et al. 2007).

Firstly, an important facilitator of sustainability appears to be the coordinators’ changed attitudes to the relevance of sex and gender in healthcare. During the implementation phase, the change agent plays a significant role in convincing others of the value of implementing gender health issues. In the long term, however, medical educators become key persons as the start-up tasks of the change agent must be taken over by the organization (Glind et al. 2015). As coordinators now acknowledge the importance of sex and gender in medicine, they also take teaching about sex and gender for granted. They are also convinced they can prevent the disappearance of sex and gender issues by discussing these issues with their fellow teachers.

Secondly, though the coordinators proved to be motivated and aware of the importance of sex and gender issues, they experience lack of time required to integrate sex and gender issues into medical education and to keep up with the expanding body of knowledge of gender health issues. As a result, medical teachers are less motivated and less involved to further develop their teaching. Implementation studies in healthcare also reported time as a factor for achieving maintenance (Glind et al. 2015; Huijg et al. 2013).

This study underlines that, in the context of medical education, implementation projects need to take into account that coordinators experience difficulties focusing on medical education while combining duties as teachers, researchers and physicians. Over and above their standard professionalism, teachers need to be given support at an organizational level to spend time on developing educational materials and to enhance their own teaching competencies in sex and gender. In a well-coordinated staff setting, teamwork amongst coordinators will promote the success and sustainability of innovations, and, conversely, the more teachers work individually, the less likely it is that innovations will be adopted (Bland et al. 2000). An open and supportive culture enhances the implementation process, which reinforces the link between educational improvements and organizational culture (Hallinger and Heck 2011; Sleegers et al. 2014).

Thirdly, the need for embedding sex and gender in objectives, examinations and evaluations in order to maintain its implementation has also been an important factor over the years. Glind et al. (2015) also describe that ongoing improvement and innovation, and dealing with fluctuating organizational policy are important factors for maintenance. In this process, revisions can be a window of opportunity to integrate sex and gender firmly into the medical curriculum, but they may also reverse good results. Remarkably, while observing that the upcoming revision was important, none of the coordinators mentioned a substantial revision that took place in 2006. At that time, sex and gender had been designated by the medical faculty as one of the six education objectives.

In our opinion, this top-down support from the medical faculty has contributed to the sustainability of gender health issues and the adjustments added over the years, as it has caused sex and gender to be adapted to the faculty’s context and future development and to be incorporated into quality control processes. As the objectives have become institutionalized, new coordinators might be unaware of previous implementation efforts and take the objectives on sex and gender, once imposed by the faculty, as self-evident. This finding suggests that implementation of sex and gender needs to be explicitly designated as an important goal by the faculty to become part of objectives, examinations and evaluations.

The introduction of Bachelor’s and Master’s programs is another reform of the medical curriculum that took place in the past decade. This reform can explain our new finding that biomedical sex-based differences tended to be retained more easily than psychosocial gender-based differences. The Bachelor’s-Master’s structure was introduced into the curriculum at the time of the 2005 implementation project. The Bachelor’s curriculum is a three-year mainly theoretical program, in which it is easier to discuss biomedical issues than psychosocial issues, which are more suited to the practice-oriented Master’s program. Therefore, psychosocial gender differences could have been rescheduled to the Master’s program. Moreover, the design of the two medical professional development courses (MPD) was also renewed, causing some gender issues to disappear or to be rescheduled to other courses.

This shift in itself does not matter as long as the Master’s courses bring psychosocial gender issues to the students’ attention, to make sure that students are aware of both the biomedical and the psychosocial aspects of gender medicine when they graduate from medical school. In order to get a full picture of the gender perspective of medical education, future research should also take the Master’s program into account.

### Implications and further research

As medical teachers are key figures in safeguarding the continuity of the implementation process, they should be engaged in the establishment of a continuous gender perspective in medical education. Teachers will be more motivated by discussing sex- and gender-related cases in small-group educational meetings (Celik et al. 2009). As research, education and care demands contribute to workload pressure and as coordinators at the Nijmegen faculty lack time to enhance their sex and gender competencies and keep up with a growing body of knowledge, future implementation projects at other faculties need to take these factors into account to achieve successful implementation of gender health issues. Further screening would allow us to explore whether these facilitating and impeding factors also exist at the other Dutch medical faculties.

Lastly, a well-formulated evaluation plan may prove to be helpful in consolidating sustainability and developing new educational materials in a structured way. To prevent the sustainability of sex and gender issues from being dependent on the decisions of individuals, a solid integration of sex and gender into the faculty could be achieved by incorporating sex and gender into curriculum objectives and evaluations. The structural embedding of sex and gender as an evaluation criterion at the national level is necessary to prevent faculties from straying into arbitrary discourses. Moreover, as our study was restricted to the Bachelor’s program, the presence of sex and gender issues in the Master’s program remains unexplored.

### Strengths and limitations

The strength of this study lies in its longitudinal evaluation of the sustainability of incorporating sex and gender into medical education, by comparing the 2014 coursebooks with those of 2005. Additional information provided by the coordinators may contribute greatly to the successful implementation of gender health issues at other medical faculties.

A limiting factor may have been our non-recording of the interviews as this might reduce the validity of our data. We decided not to record the interviews to avoid the coordinators’ reluctance to take part, and in order to be able to guarantee the validity of the field notes, therefore, we presented these to the coordinators for member checking. Another limiting factor may be our focus on sex and gender issues in eight coursebooks without paying attention to other educational formats such as oral lectures or textbooks. Previous research, however, has shown that medical textbooks tend to pay little attention to sex and gender issues (Dijkstra et al. 2008).

Lastly, as the observer in the interviews was a Professor of Gender and Women’s Health, the coordinators may have given socially desirable answers. On the other hand, her presence was of added value since she was well informed about the medical curriculum at the faculty and could ask valuable additional questions. At the time of the interviews, she had not been involved in teaching in the Bachelor’s program for 5 years.

## Conclusion

In the process of integrating sex and gender into medical education, most adjustments made in the 2005 implementation project were sustained over the past decade. As medical teachers play a pivotal role in the incorporation of sex and gender, they should be supported in being able to spend time on gender health issues in medical education and to keep their knowledge of sex and gender up to date. As medical education is a field where ongoing change is inevitable, sex and gender should be firmly embedded in learning objectives and curriculum evaluations. We advise medical staff and educators not only to pay attention to factors that facilitate implementation at the start, but also to create conditions that ensure its maintenance in the long term.
